# Bone Marrow Dominant Erdheim–Chester Disease Lacking Characteristic Radiologic Findings: A Diagnostic Pitfall

**DOI:** 10.1002/jha2.70452

**Published:** 2026-11-09

**Authors:** Elsire Boulanger Laborit, Sarisha Naidoo, Habib Moshref Razavi

**Affiliations:** ^1^ Department of Pathology and Laboratory Medicine University of British Columbia Vancouver British Columbia Canada; ^2^ Division of Hematopathology and Transfusion Medicine Department of Laboratory Medicine The Royal Columbian Hospital Fraser Health Authority New Westminster British Columbia Canada

## 1 Case Description

A 77‐year‐old woman presented with progressive weight loss, transfusion‐dependent anemia, neutropenia, and splenomegaly. Cross‐sectional imaging demonstrated marked splenomegaly but no lymphadenopathy, osteosclerotic long‐bone lesions, perirenal soft‐tissue infiltration, cardiovascular involvement, or other characteristic radiologic manifestations of Erdheim–Chester disease (ECD). Bone marrow examination demonstrated a hypercellular (approximately 70%) marrow with multilineage dysplasia, moderate reticulin and collagen fibrosis, and an interstitial histiocytic infiltrate involving approximately one‐quarter of the marrow cellularity. The histiocytes showed abundant pale cytoplasm with focal foamy changes, round‐to‐ovoid nuclei, scattered Touton‐type giant cells, and focal hemophagocytosis (Figure 1A). Immunohistochemistry demonstrated strong expression of CD68, CD163, factor XIIIa, cyclin D1, and BRAF V600E, while CD1a and Langerin were negative, and S100 showed only weak, nonspecific staining (Figure 1B,C). Conventional cytogenetic analysis demonstrated a complex karyotype compatible with an associated myeloid neoplasm, and targeted next‐generation sequencing confirmed a BRAF V600E mutation. Together, the morphologic, immunophenotypic, and molecular findings established the diagnosis of marrow‐dominant ECD [1, 2].

**FIGURE 1:**
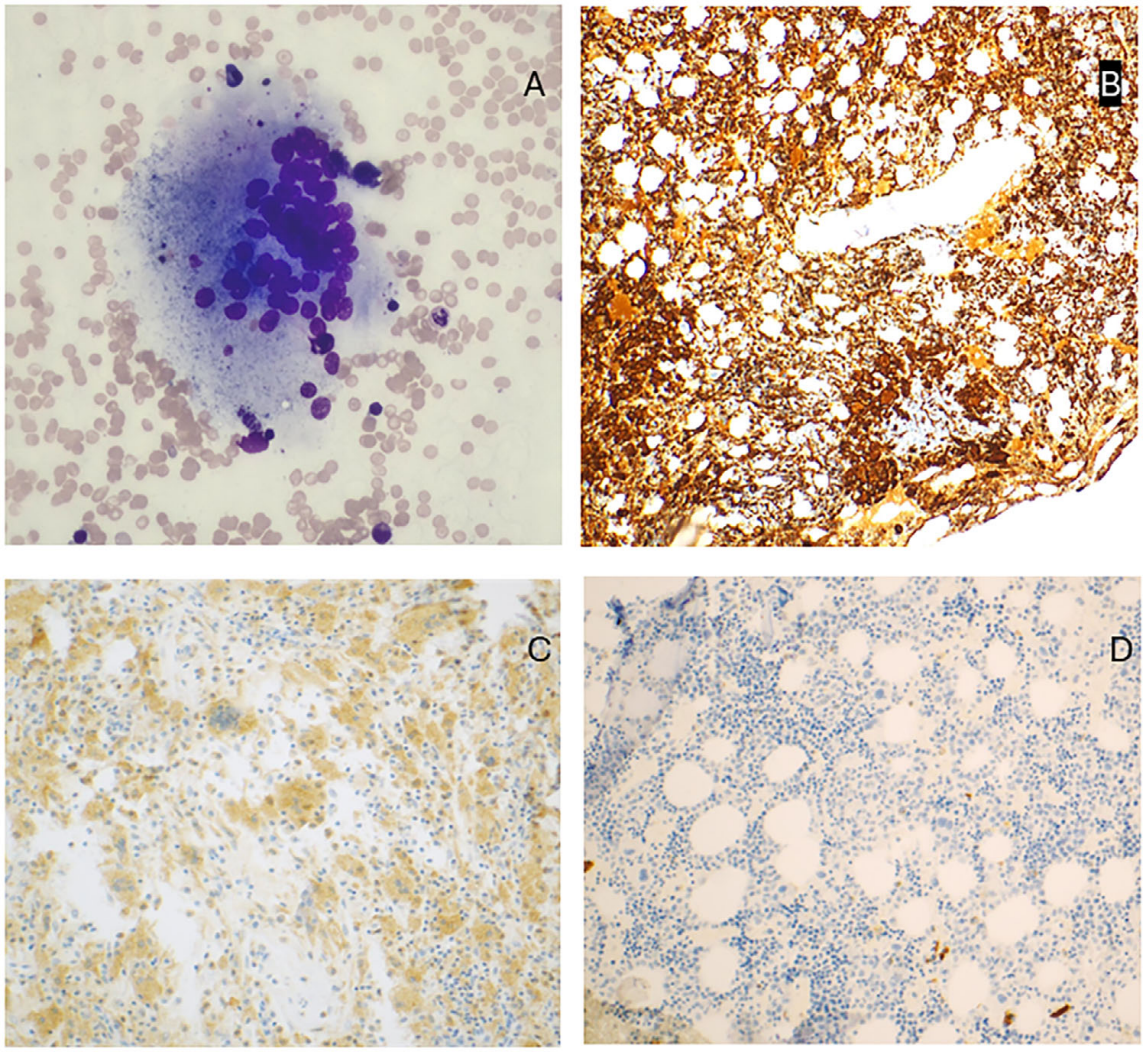
Bone marrow dominant Erdheim–Chester disease. (A) Bone marrow aspirate smear demonstrating foamy histiocytes and scattered Touton‐type giant cells (May–Grünwald–Giemsa stain, ×500). (B) CD163 immunohistochemistry highlighting the interstitial histiocytic infiltrate. (C) BRAF V600E immunohistochemistry demonstrating diffuse cytoplasmic positivity in the lesional histiocytes. (D) Absence of Langerin expression excluding Langerhans cell histiocytosis.

ECD is a clonal histiocytic neoplasm driven by constitutive activation of the MAP kinase pathway, most commonly through BRAF V600E, and is classified within the L group of histiocytoses [1, 2]. The diagnosis is frequently suggested by characteristic radiologic findings, including bilateral osteosclerotic long‐bone lesions and perirenal or cardiovascular involvement [1, 3]. In contrast, isolated or marrow‐predominant disease is distinctly uncommon and may present a substantial diagnostic challenge. In the absence of characteristic imaging findings, the differential diagnosis includes reactive histiocytosis, Langerhans cell histiocytosis, Rosai–Dorfman disease, juvenile xanthogranuloma, histiocytic sarcoma, and histiocytic proliferations associated with myeloid neoplasms. Recognition of the characteristic morphologic features together with the immunophenotype, particularly BRAF V600E positivity with absent CD1a and Langerin expression, is critical for establishing the diagnosis [1, 2]. Increasing evidence also supports a biologic association between ECD and clonal myeloid neoplasms, emphasizing the importance of integrating morphologic, immunophenotypic, cytogenetic, and molecular findings [4]. This case highlights that bone marrow examination may provide the first and only diagnostic clue to ECD when the characteristic radiologic manifestations are absent, thereby facilitating accurate diagnosis and appropriate consideration of targeted therapy.

## Funding

The authors have nothing to report.

## Conflicts of Interest

The authors declare no conflicts of interest.

## Data Availability

The data that support the findings of this study are available on request from the corresponding author. The data are not publicly available due to privacy or ethical restrictions.
